# Effect of robot-assisted training for lower limb rehabilitation on lower limb function in stroke patients: a systematic review and meta-analysis

**DOI:** 10.3389/fnhum.2025.1549379

**Published:** 2025-03-05

**Authors:** Hongyao Wang, Haifei Shen, Yi Han, Wenlu Zhou, Junjie Wang

**Affiliations:** School of Nursing, Zhejiang Chinese Medical University, Hangzhou, Zhejiang, China

**Keywords:** robot-assisted, stroke, lower limb function, rehabilitation, meta-analysis, systematic review

## Abstract

**Objective:**

The effectiveness of lower extremity rehabilitation robots in rehabilitating stroke patients is still controversial. With this systematic review, the aim is to analyze whether the assisted training of the lower limb rehabilitation robot is more effective in promoting the rehabilitation of lower limb function in stroke patients compared with traditional physical therapy.

**Methods:**

We conducted a thorough search of nine databases for relevant randomized controlled trials published between the time of their construction and February 2024. The Cochrane Collaboration tool was used to assess the risk of bias in each included literature, and meta-analyses and subgroup analyses were carried out with Revman 5.4 software. This study followed the PRIMA reporting statement provided by EQUATOR.

**Results:**

The meta-analysis includes 12 articles and 651 patients. Lower limb rehabilitation robot-assisted training significantly improved lower limb motor function, walking ability, and lower limb ability to balance in stroke patients. However, the effect on gait coordination was not statistically significant.

**Conclusion:**

Robot-assisted training for lower limb rehabilitation may considerably enhance motor function, walking capacity, and balance function in stroke patients while also providing a novel option for patients to recuperate.

**Systematic review registration:**

http://www.crd.york.ac.uk/prospero/#recordDetails, identifier CRD-42024504930.

## 1 Introduction

Stroke is a frequently occurring severe cerebrovascular disorder that leads to considerable death and disability (GBD 2019 Stroke Collaborators, 2021). The World Health Organization (WHO) states that stroke is the second most prevalent cause of death worldwide (Feigin et al., 2022). Globally, more than 15 million people have their first stroke each year, and nearly a third of them die or suffer major impairment as a result of the condition, which is particularly prevalent among the elderly (Wafa et al., 2020). Stroke often results in impaired lower limb function. According to the ICF (International Classification of Functioning, Disability and Health) framework, lower limb dysfunction is usually characterized by significant weakening of the strength of lower limb muscle groups, limited range of motion of joints, and deterioration of motor control and coordination (Leonardi et al., 2022). Previous studies have shown that about 60–70% of stroke patients have varying degrees of lower limb dysfunction, resulting in irregular gait, difficulty with postural changes, and decreased standing and balance abilities (Gorst et al., 2019; Yang et al., 2023). These dysfunctions not only impair the patients’ bodily functions, but also severely limit their independence and social participation in daily life, potentially leading to social isolation and psychological issues (Dai et al., 2024). Therefore, the recovery of lower limb function in stroke patients is critical to improving their quality of life and psychological health. Interventions for lower limb function after stroke should be carefully considered, and personalized rehabilitation treatments should be devised to help patients recover their lower limb function.

Although traditional methods of lower limb rehabilitation for stroke can help patients restore function to a certain extent, there are some degree of limitations. Especially for stroke patients with muscle weakness in the lower limbs, they are prone to fatigue during the rehabilitation process. During gait training, patients may soon be unable to continue training due to physical exhaustion, which not only reduces the effectiveness of rehabilitation, but also may lead to decreased adherence to the rehabilitation program. These factors limit the effectiveness and durability of traditional rehabilitation methods. As an emerging rehabilitation intervention, lower limb rehabilitation robots have developed rapidly in recent years. Compared with traditional physical therapy, rehabilitation robot-assisted training can provide high-precision motor control, helping patients gradually restore normal muscle strength and gait through accurate gait simulation and force regulation (Rodríguez-Fernández et al., 2021), which in turn ensures that patients engage in standardized exercise training. This accuracy can significantly improve patient rehabilitation outcomes. At the same time, traditional rehabilitation procedures often rely on the experience and intuition of therapists, which may contain some subjective errors. However, lower limb rehabilitation robots are typically outfitted with modern sensors and real-time monitoring systems (Plaza et al., 2023). These technologies can continuously track the patient’s rehabilitation process and adjust the treatment plan in time, which facilitates the implementation of correct and scientific rehabilitation treatment (Calafiore et al., 2022). Numerous studies have demonstrated that high-intensity, repetitive training aids in the recovery of motor function and brain plasticity (McCabe et al., 2015; Ward et al., 2019; Cramer et al., 2021). The ability of rehabilitation robot-assisted training to accurately deliver high-frequency, continuous, and high-intensity training helps patients recover more quickly, become more resilient, and experience less weariness and discomfort from extended treatment. More significantly, using robots gives patients a new therapy option by allowing them to self-rehabilitate at home, particularly in situations when medical resources are scarce or a therapeutic setting is unavailable (Goffredo et al., 2020; Lee et al., 2022).

Lower limb rehabilitation robots are automated rehabilitation training devices that combine rehabilitation medicine, computer science, biomechanics, artificial intelligence, and other fields to inhibit abnormal gait through repetitive simulation of normal walking patterns, improve muscle strength in the affected limbs, restore nervous system control of the limbs, and eventually recover lower limb function (Lee et al., 2022; Huang et al., 2017). Robotic devices commonly used to assist training are mainly categorized into end-effectors and exoskeletons; end-effectors are classified as pedal-based and platform-based, while exoskeletons are classified as ground-based and treadmill-based robots with weight reduction systems (Bhardwaj et al., 2021). However, most previous research either investigated the rehabilitative effects of exoskeleton robots or explored robotic treatments for the recovery of upper limb motor function in patients (Hsu et al., 2023; Chien et al., 2020), and were unable to evaluate the recovery of lower limb function in patients comprehensively. Meanwhile, the results are not consistent between different research due to significant heterogeneity in treatment procedures, training period, and frequency (Yeung et al., 2018; Louie et al., 2021). Therefore, this study aims to summarize the effectiveness of assisted training with two lower limb rehabilitation robots on lower limb dysfunction in stroke patients, as well as to determine whether the duration of different training procedures influences rehabilitation outcomes. This provides a reference basis for the early rehabilitation of clinical patients and the development of individualized training programs.

## 2 Methods

This meta-analysis was conducted according to the Preferred Reporting Items for Systematic Reviews and Meta-Analysis (PRISMA) guideline (Page et al., 2021). The study protocol was registered in PROSPER (CRD-42024504930).

### 2.1 Search strategy

The search included eight databases including PubMed, Web of Science, Cochrane Library, Embase, Weipu database, China National Knowledge Infrastructure (CNKI), WanFang, and China Biomedical Literature Database (CBM), with a search date of the build date to February 2024. A comprehensive search was performed using a combination of subject phrases and free words: “Stroke” OR “Cerebrovascular disease” “cerebral stroke” OR “Ischemic stroke” OR “Cerebral infarction” OR “Cerebrovascular accident” OR “CVA” AND “Limb function” OR “Motor function” OR “Lower Extremity function” AND “Rehabilitation Robotics” OR “Exoskeleton Device” OR “Exoskeleton robots” OR “Wearable exoskeletons.” Detailed search strategies and exclusion criteria can be found in Appendix S1.

### 2.2 Inclusion criteria

This study used the PICOS (Population, Intervention/Question of Interest, Comparison, Outcome, and Study Design) methodology to identify studies for inclusion. The eligibility criteria were as follows: (1) A randomized controlled experiment (RCT) was the study’s design; (2) Patients who were at least eighteen years old, with a confirmed diagnosis of stroke by CT or MRI, lower extremity dysfunction such as hemiparesis, muscle weakness, lower extremity ataxia, and dystonia, no limitations on the duration of the disease, with stable vital signs, clear consciousness, and no cognitive impairment or other neurological disorders; (3) Intervention: the experimental group used lower limb rehabilitation robot-assisted training or combined with traditional therapy, whereas the control group used alternative therapies, including medicines, acupuncture, and conventional rehabilitation; and (4) Outcome indicators include the lower limb section of the Fugl-Meyer Motor Function Scale (FMA-LE), the Berg Balance Scale (the BBS), the 6 min walking test (6MWT), and the Time-Up-and-Go Test (TUGT).

### 2.3 Exclusion criteria

(1) Duplicate publications; (2) preliminary experiments, reviews, or conference abstracts; (3) studies for which the full text was not available; (4) studies with incomplete data or for which data information could not be extracted; (5) publications in languages other than English or Chinese; and (6) studies that did not utilize a lower extremity rehabilitation robot or that incorporated other interventions.

### 2.4 Study selection

Two researchers independently screened the literature, extracted relevant data and cross-checked them. When the two researchers disagreed, a third researcher was consulted. Duplicate literature was excluded first. Then, the titles and abstracts of the articles were read to exclude those with irrelevant topics. Finally, the full text was reviewed and the final inclusion of the literature was determined based on the inclusion and exclusion criteria. The whole process was carried out using the literature management software EndNote X21.

The selection of studies focused on gait coordination, walking ability, balance, and lower extremity motor function; these indicators have a strong connection with the functional state of the lower extremities in stroke patients. Research presenting one or more of the pertinent indicators met the criteria for inclusion in this review. These were the results: (1) Fugl-Meyer Motor Function Scale (FMA-LE), lower extremity section; (2) Berg Balance Scale (BBS); (3) 6 min walk test (6MWT); and (4) Time-Up-and-Go Test (TUGT).

### 2.5 Quality appraisal and risk of bias assessment

Two researchers separately evaluated the quality and risk of bias critical appraisals of the studies involved using the Revised Cochrane risk of bias instrument for RCTs (RoB 2 tool) (Sterne et al., 2019), and both parties checked for discrepancies. All disputes or conflicts regarding inclusion were resolved after deliberation and agreement with a third independent reviewer. The evaluation included seven assessment items: the randomization technique, assignment concealment, blinding of study workers and subjects, blinding of study assessors, completeness of outcome data, selective reporting of findings, and other potential sources of bias. The evaluator was to decide if each of these things had a “high risk of bias,” a “low risk of bias,” or an “unclear” rating. If any item in a study is rated as “high risk,” the study will be classified as having a “high risk” of bias. If all items are rated as low risk, the study will be classified as having a “low risk” of bias. If bias is present, the study will be rated as having a “low risk” of bias. If all of the above requirements are met, the quality rating is A. If they are only moderately satisfied, they receive a B quality grade. Should they be fully unsatisfied, the quality ranking is C.

### 2.6 Data extraction

Two researchers used a general information sheet to harvest data for the study separately. Authors, publication date, nation, research participant age and sample size, frequency of treatments, interventions, and outcome indicators for the trial and control groups were among the significant components.

### 2.7 Statistical analysis

RevMan 5.4 software was utilized to carry out a meta-analysis of the included research. In this study, the outcome indicators were continuous variables, hence mean difference (MD) or standardized mean difference (SMD) were used as effect indicators, and the effect analysis statistic was the 95% confidence interval. The χ^2^ test and I^2^ index were used to assess heterogeneity. If *p* > 0.1 and I^2^ < 50%, heterogeneity between studies was considered acceptable, and a fixed-effects model was applied. If *p* ≤ 0.1 or I^2^ ≥ 50%, significant heterogeneity was found between studies, and sensitivity analysis or subgroup analysis was conducted to explore the source of heterogeneity. If the source of heterogeneity could not be identified, a random-effects model was applied. The U test (*α* = 0.05) was used to test the hypothesis, with *p* < 0.05 indicating statistical significance.

## 3 Results

### 3.1 Study selection

**Figure 1:**
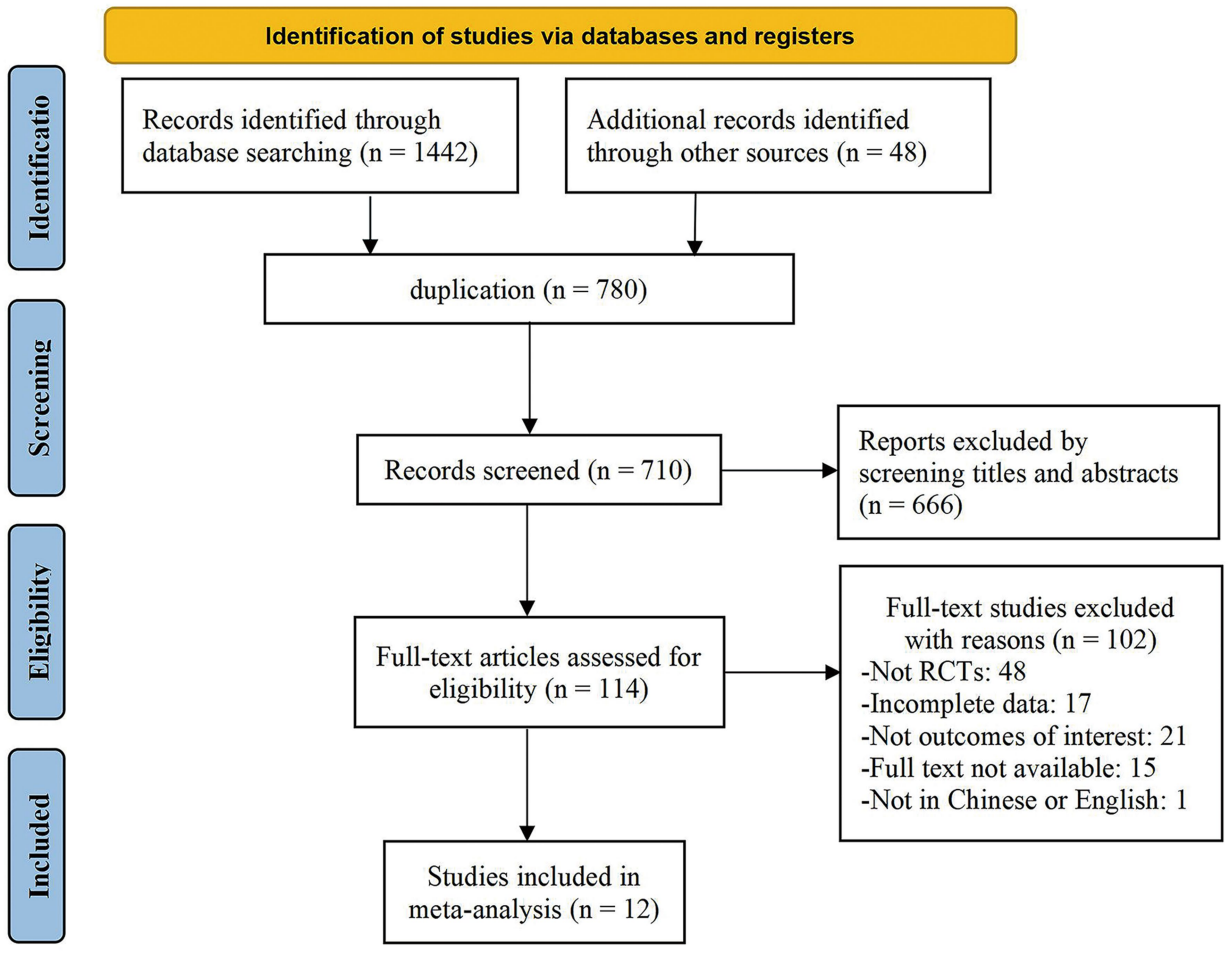
PRISMA flowchart.

The preliminary search yielded 1,490 studies. We used EndNote.21 to exclude 780 duplicates, 666 of which were irrelevant to the topic based on the title and abstract, and 102 of which were removed for various reasons after reading the whole text. Of these, 48 were not RCT trials, 17 had missing data, 15 lacked access to the full text, and 22 did not fit the inclusion requirements. Finally, this study included 12 publications (Jayaraman et al., 2019; Alingh et al., 2021; Zhang et al., 2023; Bustamante Valles et al., 2016; Nam et al., 2019; Min et al., 2020; Meng et al., 2022; Gu et al., 2020; Zheng et al., 2021; Shi et al., 2022; Le, 2020; Zhao et al., 2013). Figure 1 shows the PRISMA flowchart.

### 3.2 Characteristics of the studies

This meta-analysis included 12 RCTs published between 2013 and 2023 from four countries: the United States (*n* = 2), Netherlands (*n* = 1), South Korea (*n* = 2), and China (*n* = 7). A total of 651 patients participated, with 390 male subjects. The mean age of the participants was 54.35 years, ranging from 18 to 85 years, and the duration of stroke varied from 13.0 days to 13.3 years. Robotic-assisted training was the intervention employed in all the studies for lower limb rehabilitation; in contrast, the control group received standard rehabilitation or care. No negative effects were reported by the research that was part of this review. Table 1 provides specific study information.

**Table 1:** Characteristics of included randomized controlled trials.

Author (year)	Sample	Time since stroke (M ± SD)		Age (M ± SD)		rehabilitation robot devices	Intervention	Control	Intensity of intervention	Outcomes
	(I/C)	(I/C)		(I/C)						
Jayaraman et al. (2019)	25/25	7.1 ± 6.2 years	5.4 ± 3.0 years	59.5 ± 9.7	61.6 ± 12.6	Stride Management Assist (SMA) exoskeleton	RAT	Functional gait training with intensity-matching	45 min/d, 3d/w, 6–8w	FMA-LE BBS 6MWT
Alingh et al. (2021)	17/15	5.4 ± 1.8 weeks	5.9 ± 2.1 weeks	60.6 ± 9.3	56.8 ± 9.8	AANmDOF robotic	RAT+ CRT	CRT	30 min/d, 3d/w, 6w	FMA-LE BBS TUGT
Zhang et al. (2023)	18/16	2.50 ± 4.00 months	3.50 ± 3.00 months	56.88 ± 10.99	60.81 ± 9.61	MANBUZHEKANGFU	RAT	CRT	30 min/d, 5d/w, 4w	FMA-LE 6MWT
Bustamante Valles et al. (2016)	10/10	/	/	44.1 ± 11.90	64.1 ± 7.96	Walkbot robotic	RAT	Routine care	2 h/d, 6–8w	FMA-LE 6MWT TUGT
Nam et al. (2019)	18/16	530.11 ± 389.21 days	284.81 ± 309.04 days	48.33 ± 15.56	68.56 ± 17.35	Exowalk	RAT	CRT	30 min/d, 5d/w, 4w	FMA-LE 6MWT
Min et al. (2020)	19/19	921.52 ± 1762.00 days	788.73 ± 999.24 days	61.47 ± 11.15	56.36 ± 9.16	trunk stabilization training robot (3DBT-33)	RAT+ CRT	CRT	30 min/d, 5d/w, 4w	FMA-LE BBS TUGT
Meng et al. (2022)	62/64/61	/	/	/	/	Walkbot robotic	RAT	ELLT group: matching the intensity and duration of RAGT CRT group	45 min/d, 3d/w,4w	FMA-LE
Gu et al. (2020)	20/20	27.2 ± 7.8 days	29.4 ± 8.9 days	54.5 ± 12.7	57.6 ± 10.3	Natural Gait pelvic-assisted lower limb rehabilitation robot training	RAT+ CRT	CRT	20 min/d, 6d/w, 8w	FMA-LE BBS
Zheng et al. (2021)	20/20	13.35 ± 0.93 days	13.05 ± 0.76 days	65.5 ± 5.1	63.3 ± 6.8	Natural Gait pelvic-assisted lower limb rehabilitation robot training	RAT+ CRT	CRT	20 min/d, 5d/w, 4w	FMA-LE BBS
Shi et al. (2022)	20/20	14.55 ± 2.01 days	14.25 ± 2.19 days	68.15 ± 4.17	66.65 ± 4.79	Natural Gait pelvic-assisted lower limb rehabilitation robot training	RAT+ CRT	CRT	20 min/d, 5d/w, 4w	FMA-LE
Le (2020)	48/48	65.74 ± 18.61 days	62.37 ± 15.27 days	59.11 ± 13.45	58.24 ± 12.31	Exoskeleton robot	RAT	CRT	30 min/d, 5d/w, 6w	FMA-LE BBS
Zhao et al. (2013)	20/20	21.93 ± 1.33 days	21.13 ± 1.26 days	51.8 ± 10.14	57.6 ± 8.22	Lokomat	RAT	CRT	30 min/d, 3d/w, 10w	FMA-LE

I/C, intervention group/control group; ELLT group, enhanced lower limb treatment group; RAT, robot-assisted training; CRT, conventional rehabilitation treatment; RAGT, robotic-assisted gait training; FMA-LE, Fugl-Meyer assessment-lower extremity; BBS: Berg Balance Scale; 6MWT, Six-Minute Walk Test; TUGT: Timed Up-and-Go Test.

### 3.3 Risk of bias

**Figure 2:**
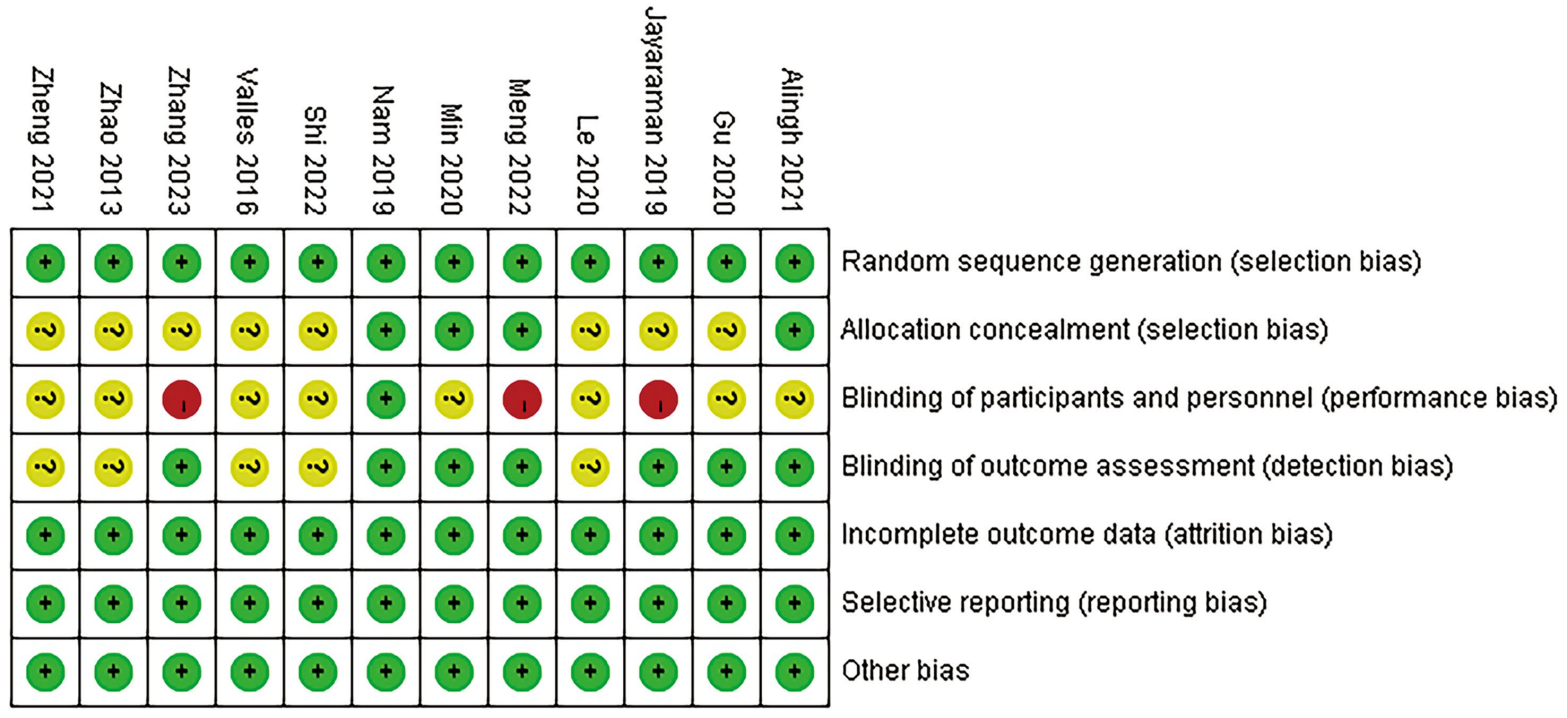
Risk of bias summary.

**Figure 3:**
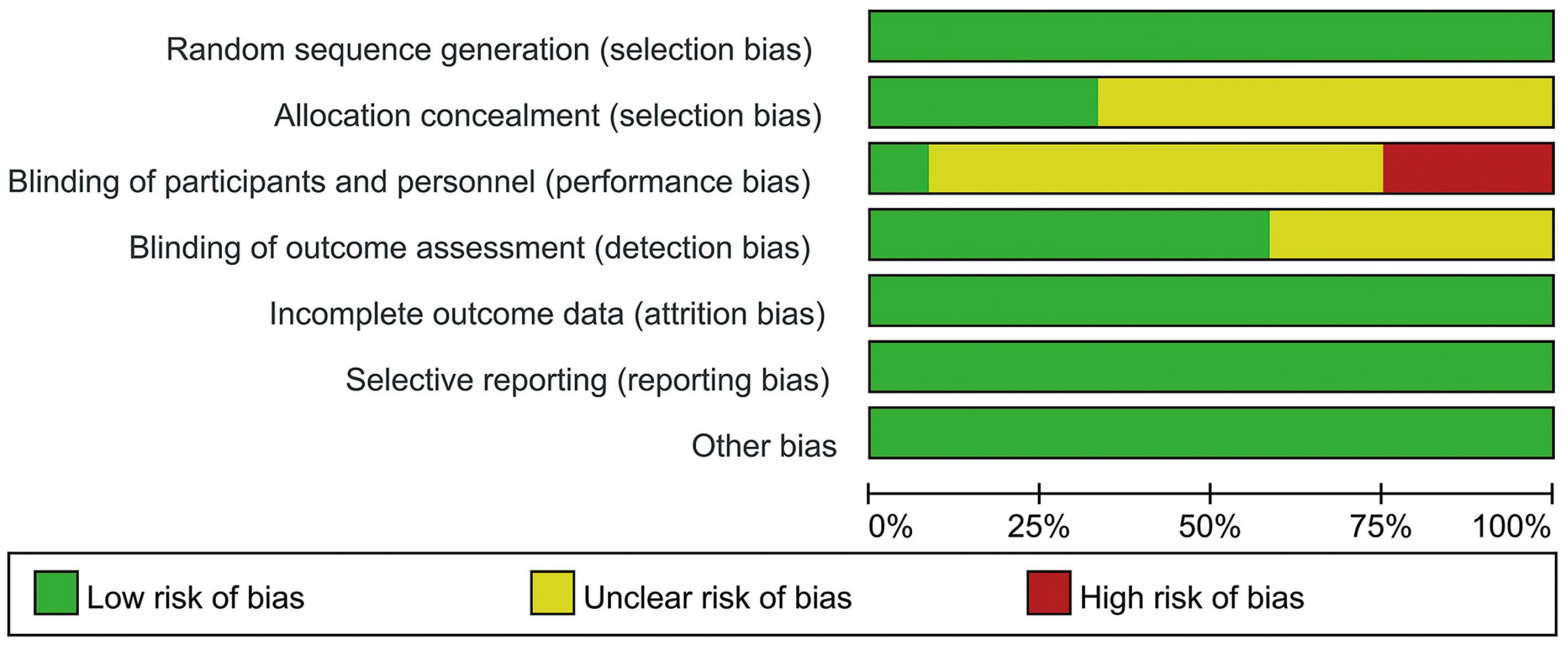
Risk of bias graph.

The meta-analysis includes 12 trials that were randomized controlled. Every trial that was included employed randomized allocation; 4 studies described the concealment of allocation, and 7 studies documented the blinding of assessors. Double-blinding proved challenging because of interference. One piece of literature was graded as A, while the rest received a B. The quality of the literature was graded as A, with the rest receiving a B. Figures 2, 3 show detailed data on the risk of bias in this study.

### 3.4 Outcomes

#### 3.4.1 FMA-LE

**Figure 4:**
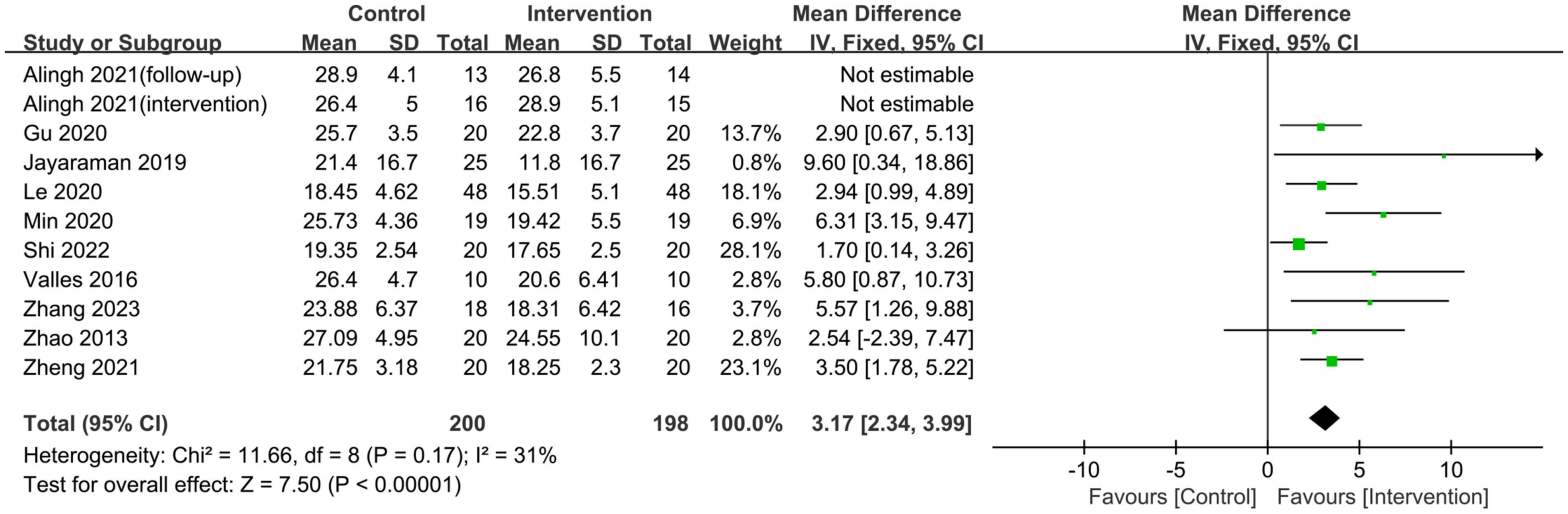
Forest plots of effect on FMA-LE.

**Figure 5:**
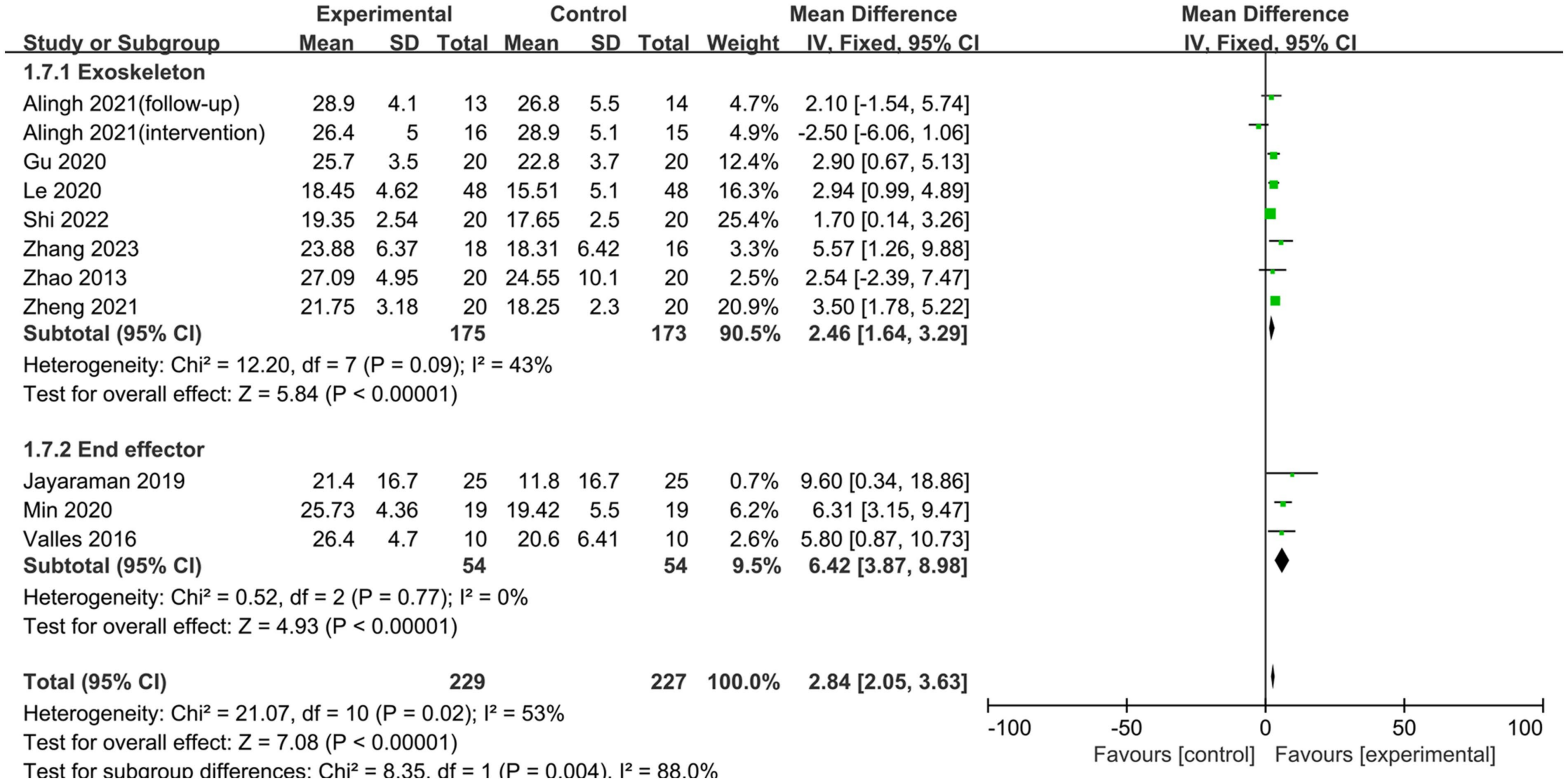
Subgroup analysis of the effect of different rehabilitation robot types on lower limb motor function in stroke patients.

Ten studies employed the FMA-LE score to assess patients’ lower limb motor function. Heterogeneity was observed in the studies (*p* = 0.02, I^2^ = 53%). After performing a sensitivity analysis to exclude the literature one by one, the results revealed that the study by Alingh et al. (2021) was the predominant source of heterogeneity. After excluding this trial, the heterogeneity test indicated no statistically significant inter-study heterogeneity (*p* = 0.17, I^2^ = 31%). A fixed-effects model was utilized in the meta-analysis. Lower limb rehabilitation robot-assisted training enhanced motor function in stroke patients, including reflex activity, muscle synergism, activity with synergism, and activity out of synergism. The difference was statistically significant [MD = 3.17, 95% CI (2.34, 3.99), *p* < 0.00001] (Figure 4). Meanwhile, we conducted a subgroup analysis based on the type of robotic device used. The results indicated that end-effector training was more effective than exoskeleton robots in improving lower limb motor function in stroke patients, with statistically significant differences [MD = 2.41, 95% CI (1.22, 3.60), *p* < 0.0001], [MD = 6.42, 95% CI (3.87, 8.98), *p* < 0.0001] (Figure 5).

#### 3.4.2 6MWT

**Figure 6:**
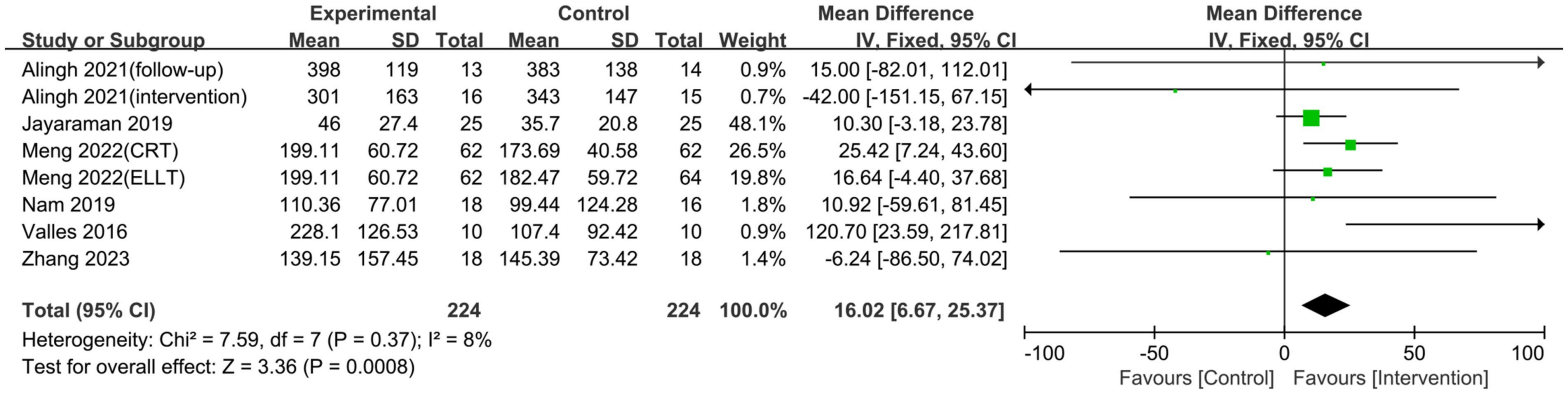
Forest plots of effect on 6MWT.

**Figure 7:**
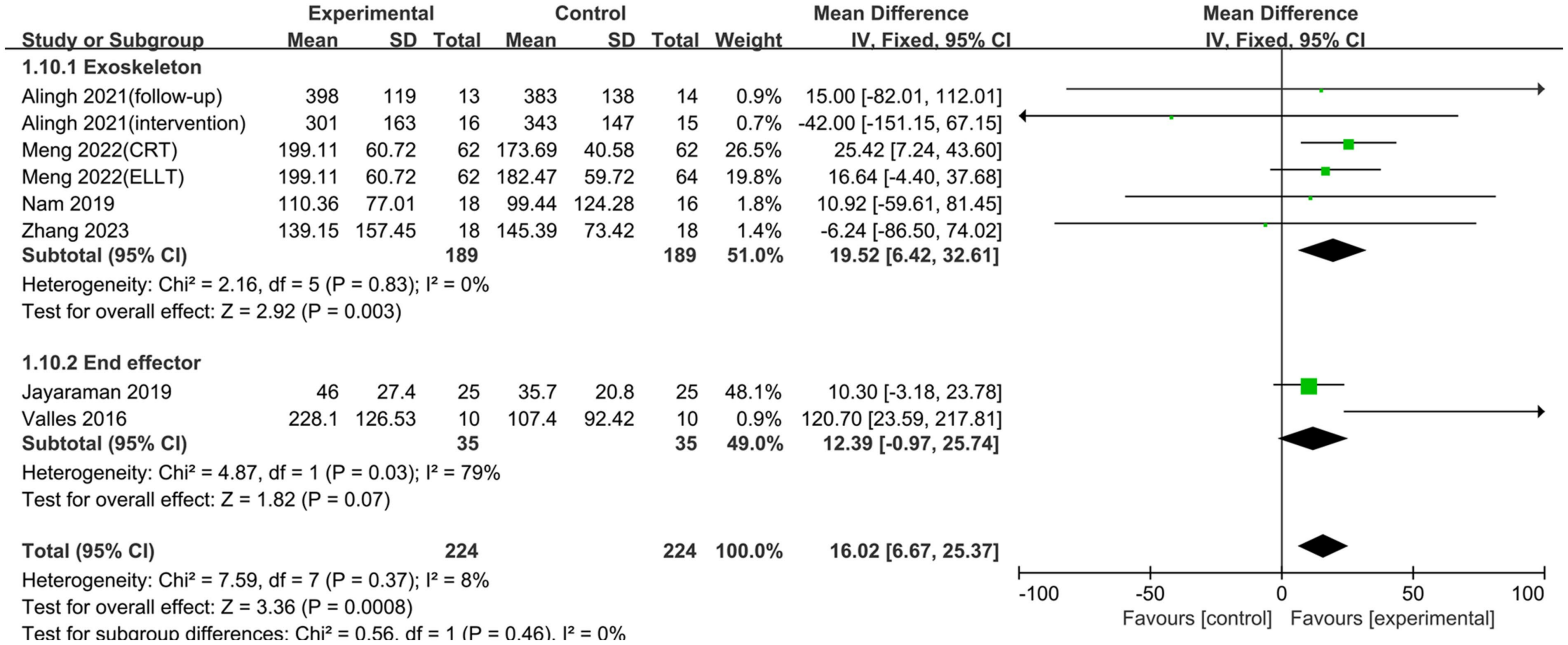
Subgroup analysis of the effect of different rehabilitation robot types on walking ability in stroke patients.

Six studies reported on the results of the 6MWT, which evaluates how far a patient can walk at the fastest feasible speed in 6 min and is commonly used to assess walking performance in stroke patients (Cheng et al., 2020). The studies had no heterogeneity (*p* = 0.38, I^2^ = 7%), hence a fixed-effects model was chosen for the meta-analysis. The study discovered that lower limb rehabilitation robot-assisted training considerably enhanced walking ability in stroke patients, with the difference being statistically significant [MD = 13.32, 95% CI (5.64, 21.00), *p* = 0.0007] (Figure 6). Our subgroup analysis showed that different robotic devices had varying effects. The exoskeleton robot was effective in improving patients’ walking function [MD = 19.52, 95% CI (6.42, 32.61), *p* = 0.003]. In contrast, the end-effector had no significant impact on walking ability [MD = 12.39, 95% CI (−0.97, 25.74), *p* = 0.07] (Figure 7).

#### 3.4.3 BBS

**Figure 8:**
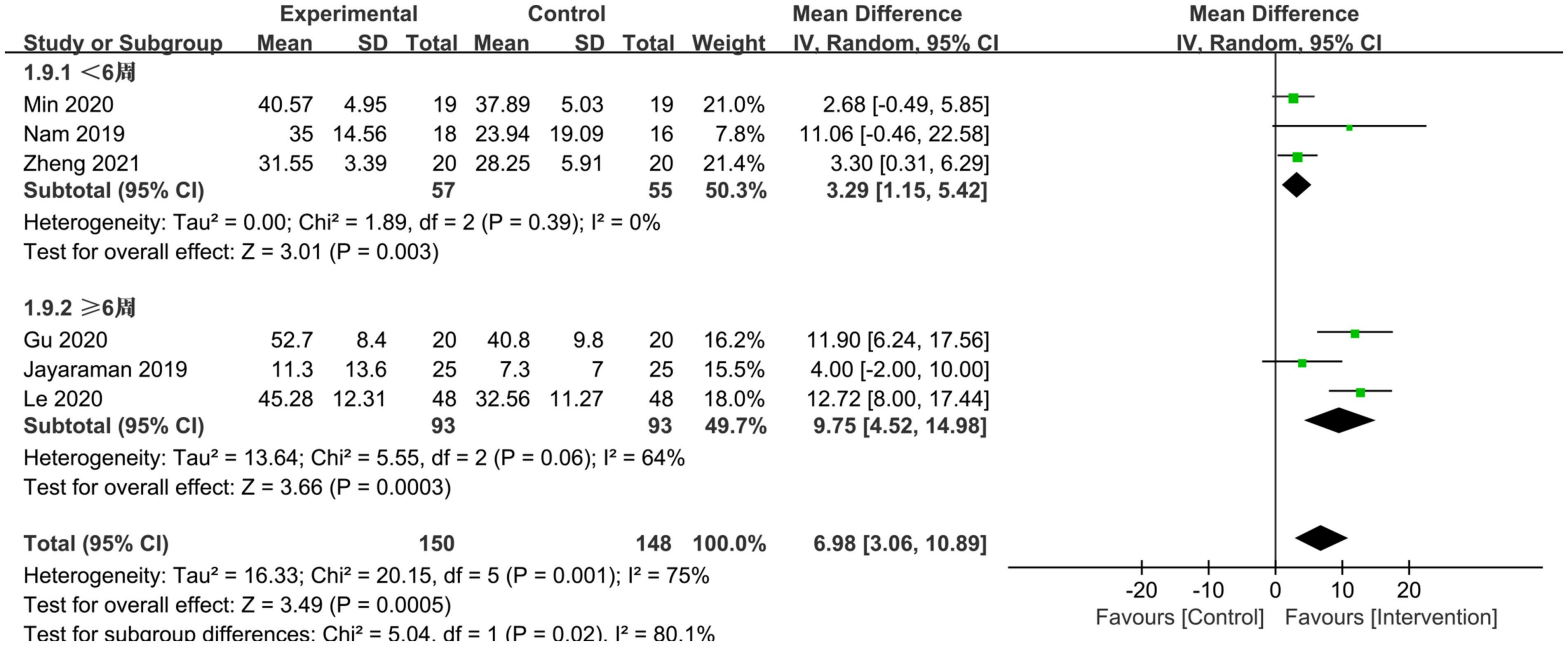
Subgroup analysis based on length of intervention.

**Figure 9:**
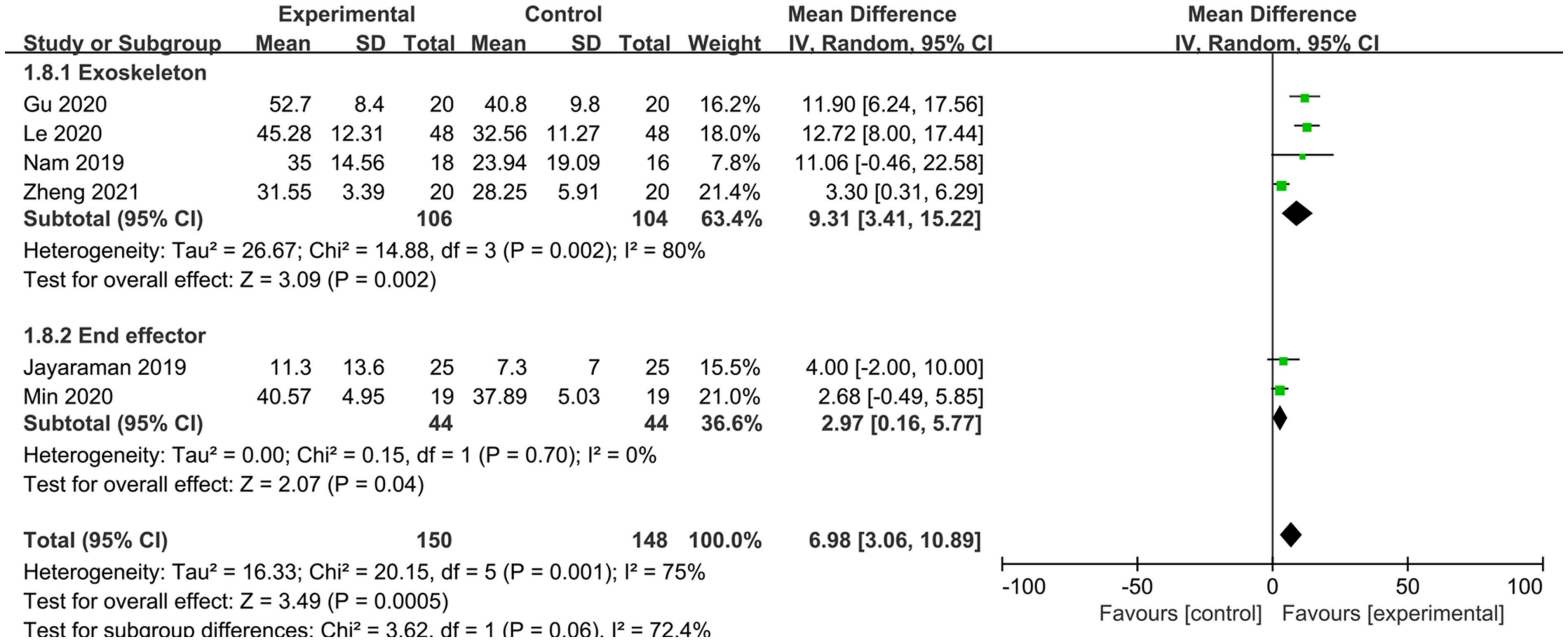
Subgroup analysis of the effect of different rehabilitation robot types on balance ability in stroke patients.

Six RCTs reported BBS scores, which varied significantly between studies (*p* = 0.0001, I^2^ = 75%). Six papers underwent sensitivity and subgroup analyses with intervention time as a criterion, but heterogeneity remained unabated, necessitating the employment of a model with random effects for meta-analysis. The results demonstrated that, when compared to conventional gait training, the lower limb rehabilitation robot successfully improved the lower limb balance function of stroke survivors, with a statistically significant difference [MD = 6.98, 95% CI (3.06, 10.98), *p* = 0.0005]. Regardless of intervention time (<6 weeks or ≥ 6 weeks), the robot group outperformed the typical rehabilitation intervention group in terms of BBS scores. Lower limb rehabilitation robot-assisted training was effective in improving the balance function of individuals suffering from stroke, with statistically significant differences [MD = 3.92, 95% CI (1.15, 5.42), *p* = 0.003], [MD = 9.75, 95% CI (4.52, 14.98), *p* = 0.0003] (Figure 8). Additionally, we conducted a subgroup analysis based on the type of robotic device used. The results showed that the exoskeleton robot was more effective than the end-effector in improving patients’ balance function, with statistically significant differences [MD = 9.31, 95% CI (3.41, 15.22), *p* = 0.002], [MD = 2.97, 95% CI (0.16, 5.77), *p* = 0.04] (Figure 9).

#### 3.4.4 TUGT

**Figure 10:**
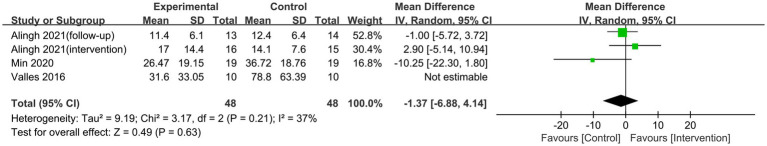
Forest plots of effect on TUGT.

Three studies assessed the TUGT score, which is used to measure lower limb gait coordination (Lorefice et al., 2017). Heterogeneity was found between studies (*p* = 0.06; I^2^ = 59%). A sensitivity analysis of three investigations found that the research conducted by Bustamante Valles et al. (2016) was the main source of heterogeneity. After excluding this trial, the heterogeneity test indicated no significant heterogeneity between studies (*p* = 0.21, I^2^ = 37%). Meta-analysis employing a fixed-effects model revealed no statistically significant difference between the two groups [MD = −1.05, 95% CI (−4.90, 2.80), *p* = 0.59] (Figure 10).

## 4 Discussion

Current studies lack a comprehensive evaluation of the different types of lower limb rehabilitation robots. Therefore, this study includes various types of rehabilitation robots to conduct a more in-depth analysis of the effects of robot-assisted training on the lower limb function of stroke patients. We found that lower limb rehabilitation robot-assisted training is more effective than traditional rehabilitation or standard care in improving stroke patients’ lower limb motor function, walking ability, and balance. Overall, robot-assisted training can improve patients’ muscle strength and postural control, and expand joint range of motion. However, the results of this study show that there is no significant difference in the effect of robot-assisted training on gait coordination in the lower extremities of stroke patients compared to traditional therapy. In addition, significant heterogeneity was observed among the studies included in this meta-analysis, primarily due to variations in treatment methods, patient characteristics, and intervention durations. These factors could impact the generalizability of the treatment effect. Therefore, the findings of this study may require further validation, depending on the patient population and treatment conditions.

We conducted a subgroup analysis of two types of rehabilitation robots and found that end-effector and exoskeleton robots showed different effects on various rehabilitation outcomes in stroke patients. According to the results of this study, the end-effector was more effective than the exoskeleton robot in improving lower limb motor function, while the exoskeleton robot demonstrated superior performance in enhancing patients’ balance. Notably, the exoskeleton robot was significantly more effective in improving walking ability, whereas the end-effector failed to show similar results. This difference may be closely related to the structural characteristics of the two types of robots. The exoskeleton robot provides external support and power by stabilizing the patient’s lower limbs and joints, which helps to restore lower limb strength, balance, and coordination (Neves et al., 2023). This not only plays an important role in improving walking ability but also assists patients in maintaining balance while standing and walking. In contrast, the end-effector robot typically improves lower limb movement patterns through precise control of foot pedal movements and repetitive training (Donnellan-Fernandez et al., 2022). However, it may have limitations in enhancing lower limb strength, postural control, and overall balance.

When a stroke occurs, the patient’s higher central nervous system loses control of the limbs due to damage to the brain nerves, resulting in lower limb motor dysfunction, such as abnormal muscle tone and proprioceptive disorders (Gao et al., 2022). Our study found that lower limb rehabilitation robot-assisted training improves the motor function of those with stroke. The neurologically significant mechanisms are explained below: Rehabilitation exercise can stimulate the paretic limb, generate new neural connections, and remodel neural circuits in the tissues around the lesion and the damaged hemisphere, as well as opposite brain, subcortical, and nerve root areas, increasing neural control over muscles (Molteni et al., 2018). During rehabilitation activities, healthcare professionals can use the lower limb rehabilitation robot to provide strong-matched and task-oriented rehabilitation training, repeatedly delivering lower limb load stimulation and positive feedback. This high-frequency, repetitive training can improve the patient’s neurological plasticity and promote lower limb motor recovery (Chen et al., 2023). In the literature evaluated in this paper, researchers use two types of lower limb robots for assisted training. Among them, the exoskeleton robot’s weight-reducing structure can reduce the contraction load of the relevant muscle groups in the lower limbs, improve muscle contraction synergy, and alleviate muscle spasms (Yao et al., 2021). Its intelligent feedback system can coordinate sensory and motor information during the training process, form a correct sensing-motor circuit, and increase the patient’s lower limbs’ proprioceptive data. The end-effector can increase the motor excitability of the patient’s cerebral cortex, improve the conductivity of the hip and knee muscles, and, ultimately, improve the lower limb motor function of stroke patients using various training methods such as obstacles, stair climbing, and ramps (Seo and Kim, 2021).

Independent walking is one of the key goals of stroke recovery, and speed and distance are commonly used to assess walking capacity (Selves et al., 2020). Some researchers have noted that lower limb muscle strength, particularly ankle joint muscle strength, is directly associated with walking ability, and ankle muscular weakness slows human walking speed (Cho et al., 2021). This meta-analysis found that lower limb rehabilitation robot-assisted training can considerably enhance stroke patients’ walking abilities, which is similar to Lee’s findings (Lee et al., 2023). The possible mechanism is that robot-assisted training enhances walking ability through multiple pathways, especially by improving ankle joint function. During training, the healthcare professional applies an ankle-foot orthosis or immobilizes the ankle joint to improve muscle strength and flexibility by improving ankle dorsiflexion function and relieving muscle spasms (Choo and Chang, 2021; Johnson et al., 2023). Improved ankle function not only improves gait stability, but also increases the patient’s walking speed. In addition, during assisted training, the rehabilitation robot can precisely control the flexion and extension of the hip and knee joints to ensure the coordinated linkage of these joints (Miyagawa et al., 2023). This high-precision motion control can give patients with consistent and standardized training, decrease human error, and compensate for traditional rehabilitation training deficiencies. With this mechanism, patients can walk for longer periods while also triggering adaptive changes in the nervous system to improve walking endurance. In Jayaraman’s study, stroke patients were followed up for 3 months, and the results showed continued improvements in walking endurance and speed in the group using the SMA exoskeleton intervention. This further suggests that lower extremity rehabilitation robots can not only facilitate the recovery of walking function in stroke patients but also yield long-term positive effects.

Stroke patients with hemiplegia exhibit symptoms such as shorter standing times on the affected side and a center of gravity tilt toward the healthy side (Yamamoto et al., 2022). Patients’ balance function suffers as a result of the unequal distribution of muscular force, increasing their risk of falling and, in extreme cases, losing the ability to care for themselves in daily life (Kim et al., 2019). This meta-analysis found that lower limb rehabilitation robot-assisted training can considerably enhance the lower limb balance function of stroke patients, particularly in terms of stabilizing and controlling the body’s center of gravity. The recovery of balance function not only depends on the improvement of lower limb muscle strength, but also on the coordination of multiple factors such as trunk control, pelvic stability, and center of gravity transfer (Westlake and Patten, 2009; Lyu et al., 2023). Lower limb rehabilitation robots can effectively improve the stability of the pelvis and hip joints through trunk stability training, thereby stabilizing the center of gravity and balance of the human body and reducing the risk of falls (Wiśniowska-Szurlej et al., 2023; Coenen et al., 2012). At the same time, a clinical trial conducted by Haruyama et al. found that balance was improved by enhancing the patient’s core and trunk stability (Haruyama et al., 2017). Furthermore, Min et al. (2020) used a rehabilitation robot to design a method of training through gamification, training patients’ trunk stability through activities such as grabbing fruit and controlling balloon bursts. The addition of weight sensors on the foot pedals significantly enhanced patients’ balance, aiding them in effectively shifting their center of gravity while strengthening and coordinating their lower limbs (Askim et al., 2018). This interactive training approach transforms the traditional “passive treatment” model of rehabilitation by allowing patients to engage in exercises within a more comfortable environment. By increasing patient involvement, it not only improves adherence but also boosts motivation, leading to better overall rehabilitation outcomes.

The subgroup analysis by intervention course revealed that the effect size of ≥6 weeks of intervention utilizing lower limb rehabilitation robot-assisted training was larger, indicating that robot-assisted training has a long-term effect on the balance function of stroke patients. The reasons behind this are as follows: (1) the rehabilitation robot may give focused, rich information and training, which promotes the patient’s active engagement; (2) Robot-assisted training is repetitive and regular, and extended intervention durations can cause relevant brain neural remodeling frequently and repeatedly, resulting in a stronger rehabilitative effect (Wang et al., 2023). Simultaneously, healthcare practitioners should tailor the exercise program for stroke patients, choose the suitable intervention frequency determined by the patient’s tolerance level, and progressively raise the training intensity to improve lower limb balance.

According to this meta-analysis, there was no discernible improvement in gait coordination among stroke patients using the lower limb rehabilitation robot. This could be due to several factors. First, the analysis included only two papers, which increases the limitations of the research results and increases the risk of false negative results. Second, there is evidence (Wiśniowska-Szurlej et al., 2023) that lower limb rehabilitation robots, after training, can accurately train lower limb joints by flexing and extending the ankle, knee, and hip joints and the patient’s pelvic translation, so that patients can recover from abnormal gait. In contrast, Bustamante Valles et al. (2016) focused their intervention on three types of postural control training: sitting, standing, and standing to sit. Therefore, to improve the effectiveness of rehabilitation activities, healthcare professionals using lower limb rehabilitation robots to improve gait coordination in stroke patients should focus on the linkage of the ankle, knee, hip, and pelvis.

## 5 Strengths and limitations

This meta-analysis is significant since it is the first to assess the efficacy of robotic-assisted training for lower limb rehabilitation on functional rehabilitation in those suffering from stroke. Furthermore, the conclusions of this research may aid in the prognosis and rehabilitation of stroke patients’ lower limbs, as well as serve as the foundation for future clinical stroke patient care and rehabilitation. However, this study has certain drawbacks. First, there was less high-quality literature and a literature rating of B for the majority of the studies that were included in this study. Second, the intervention design did not allow for double-blinding, and most studies did not clarify the allocation concealment mechanism, which could lead to selection bias. Moreover, the small sample size in each study may contribute to potential bias. The source of variability may be strengthened by incorporating studies from different countries and regions with inconsistent intervention timing and protocols. Finally, only English-language literature was included, which could introduce language bias.

## 6 Implications for future research

(1) More detailed and rigorous study protocols should be devised, including trial grouping methods, assessment indexes, and whether to allocate concealment and follow-up time. (2) Continuous study, development, and optimization of the technology of the lower limb rehabilitation robot; some studies have found that simulator syndrome arises in the elderly during training, and increasing the robot’s comfort and safety requires additional discussion. (3) In addition to focusing on short-term rehabilitation outcomes, researchers should think about the role of lower limb rehabilitation robots in long-term stroke therapy. The impact of robotic therapy on patients’ long-term walking abilities and quality of life should be evaluated by extending the follow-up period. (4) Encourage the translation of clinical trial findings into practical applications, as well as the widespread application of lower-limb rehabilitation robots in stroke survivors’ functional rehabilitation. Meanwhile, clinical healthcare staff should receive additional training and instruction to increase their knowledge and operation skills with lower limb rehabilitation robotics.

## 7 Conclusion

In conclusion, the results of this meta-analysis demonstrate that robot-assisted training significantly improves lower limb function in stroke patients, particularly in enhancing motor, walking, and balance abilities. These findings offer a strong foundation for clinical rehabilitation practices, highlighting that robot-assisted training, as a complement to traditional rehabilitation methods, can enhance rehabilitation outcomes for stroke patients. However, despite the evident clinical benefits, further research is required to explore strategies for broader application of this technology in clinical settings, as well as to address challenges such as its high cost and operational complexity. Additionally, future multi-center, high-quality, large-sample clinical research will be needed to confirm the findings and provide a more reliable basis for treating and rehabilitating lower limb function in stroke survivors.

## Data Availability

The original contributions presented in the study are included in the article/Supplementary material, further inquiries can be directed to the corresponding author.
