# Supplementary material for: Effect of robot-assisted training for lower limb rehabilitation on lower limb function in stroke patients: a systematic review and meta-analysis
Source: Front Hum Neurosci. 2025 Mar 5;19:1549379. doi: 10.3389/fnhum.2025.1549379 (PMC11919835; doi:10.3389/fnhum.2025.1549379)
Supplement: Supplementary file 1 [file Table_1.docx]

**search strategy**

| Stroke | Lower extremity function | Rehabilitation robotic |
| --- | --- | --- |
| PubMed:  (((((((Stroke) OR (Cerebrovascular disease)) OR (cerebral stroke)) OR (ischemic stroke)) OR (Cerebral infarction))OR (Cerebrovascular accident)) OR (CVA)) | PubMed:  (((Motor function) OR (Limb function)) OR ((Lower Extremity) AND (function))) | PubMed:  (((((Rehabilitation) AND (Robotics)) OR (Exoskeleton Device)) OR (Exoskeleton robots)) OR (Wearable exoskeletons)) |
| Embase:  cerebrovascular accident/exp OR 'cerebrovascular accident' OR 'stroke':ab,ti OR 'cerebrovascular disease':ab,ti OR 'cerebral stroke':ab,ti OR "ischemic stroke':ab,ti OR 'cerebral infarction':ab,ti OR 'cerebrovascularaccident':ab,ti OR 'cva':ab,ti | Embase:  'lower limb'/exp OR 'lower limb' AND 'function':ab,ti OR 'limb function':ab,ti OR 'motor function':ab,ti | Embase:  'rehabilitation'/exp AND 'robotics'/exp OR 'exoskeleton device':ab,ti OR 'exoskeleton robots':ab,ti OR 'wearable exoskeletons':ab,ti |
| Cochrane Library:  Stroke[Mesh] OR (Cerebrovascular disease:ti,ab,kw OR (cerebral stroke):ti,ab,kw OR (ischemic stroke):ti,ab,kw OR (Cerebral infarction):ti,ab,kw OR (Cerebrovascular accident): ti,ab,kw | Cochrane Library:  Lower Extremity[Mesh] AND (function):ti,ab,kw OR (Limb function):ti,ab,kw OR (Motor function):ti,ab,kw | Cochrane Library:  Rehabilitation[Mesh] AND Robotics[Mesh] OR (Exoskeleton Device):ti,ab,kw OR (Exoskeleton robots):ti,ab,kw OR (Wearable exoskeletons):ti,ab,kw |
| Web of Science:  ((((((TS=(Stroke)) OR TS=(Cerebrovascular disease)) OR TS=(cerebral stroke)) OR TS=(ischemic stroke)) OR TS=(Cerebral infarction)) OR TS=(Cerebrovascular accident)) OR TS=(CVA) | Web of Science:  ((TS=(Lower Extremity)) AND TS=(function)) OR (TS=(Limb function)) OR TS=(Motor function) | Web of Science:  ((TS=(Rehabilitation)) AND TS=(Robotics)) OR ((TS=(Exoskeleton Device)) ORTS=(Exoskeleton robots)) OR TS=(Wearable exoskeletons) |
| China National Knowledge Infrastructure (CNKI):  (((((((Stroke) OR (Cerebrovascular disease)) OR (cerebral stroke)) OR (ischemic stroke)) OR (Cerebral infarction))OR (Cerebrovascular accident)) OR (CVA)) | CNKI:  (((Motor function) OR (Limb function)) OR ((Lower Extremity) AND (function))) | CNKI:  (((((Rehabilitation) AND (Robotics)) OR (Exoskeleton Device)) OR (Exoskeleton robots)) OR (Wearable exoskeletons)) |
| WanFang:  ((((((TS=(Stroke)) OR TS=(Cerebrovascular disease)) OR TS=(cerebral stroke)) OR TS=(ischemic stroke)) OR TS=(Cerebral infarction)) OR TS=(Cerebrovascular accident)) OR TS=(CVA) | WanFang:  ((TS=(Lower Extremity)) AND TS=(function)) OR (TS=(Limb function)) OR TS=(Motor function) | WanFang:  ((TS=(Rehabilitation)) AND TS=(Robotics)) OR ((TS=(Exoskeleton Device)) ORTS=(Exoskeleton robots)) OR TS=(Wearable exoskeletons) |
| China Biomedical Literature Database (CBM):  (((((((Stroke)[Mesh] OR (Cerebrovascular disease)) OR (cerebral stroke)) OR (ischemic stroke)) OR (Cerebral infarction))OR (Cerebrovascular accident)) OR (CVA)) | CBM:  (((Motor function) OR (Limb function)) OR ((Lower Extremity)[Mesh] AND (function))) | CBM:  (((((Rehabilitation)[Mesh] AND (Robotics)[Mesh]) OR (Exoskeleton Device)) OR (Exoskeleton robots)) OR (Wearable exoskeletons)) |
| Weipu database:  (M= Stroke OR (Cerebrovascular disease) OR (cerebral stroke) OR (ischemic stroke) OR (Cerebral infarction) OR (Cerebrovascular accident) OR (CVA)) | Weipu database:  (M= (Lower Extremity function) OR (Limb function) OR (Motor function)) | Weipu database:  (M= (Rehabilitation robotics) OR (Exoskeleton Device) OR (Exoskeleton robots) OR (Wearable exoskeletons)) |

Note: In Wanfang, TS indicates Topic; In Weipu database, M indicates Topic.
